# *Simultant*: simultaneous curve fitting of functions and differential equations using analytical gradient calculations

**DOI:** 10.1186/s12859-022-04728-5

**Published:** 2022-05-21

**Authors:** Julius B. Kirkegaard

**Affiliations:** Niels Bohr Institute, University of Copenhagen, 2100 Copenhagen, Denmark

**Keywords:** Data analysis, Simultaneous fitting, Global fitting, Parameter sharing, Differential equations

## Abstract

**Background:**

The initial step in comparing mathematical models to experimental data is to do a fit. This process can be complicated when either the mathematical models are not analytically solvable (e.g. because of nonlinear differential equations) or when the relation between data and models is complex (e.g. when some fitting parameters must be shared between many data sets).

**Results:**

We introduce *Simultant*, a software package that allows complex fitting setups to be easily defined using a simple graphical user interface. Fitting functions can be defined directly as mathematical expressions or indirectly as the solution to specified ordinary differential equations. Analytical gradients of these functions, including the solution of differential equations, are automatically calculated to provide fast fitting even for functions with many parameters. The software enables easy definition of complex fitting setups in which parameters can be shared across both data sets and models to allow simultaneous fits to be performed.

**Conclusions:**

*Simultant* exploits differentiable programming and simplifies modern fitting approaches in a unified graphical interface.

## Background

Fitting mathematical functions to data can be a simple endeavor as modern computer software has made this a technically trivial operation in uncomplicated cases. However, collaborations between biologists and theoreticians have begun to strain this simplicity. Increasingly complex mathematical models are being developed and applied to biological data, and such models cannot always be represented by a simple, closed-form mathematical expression For instance, the result of mathematical modeling could be a specification of an ordinary differential equation, but not its solution.

For example, the equations describing nerve signal excitation and conduction [1] has no analytical solution. Many kinetic growth models of microorganism tend to be highly non-linear and do not permit analytical solutions [2, 3]. Likewise, models of gene expression [4], transcription networks [5, 6], enzyme kinetics [7], and a host of other biological systems follow this trend. Thus, if experimental data is to be directly compared a theoretical model, the fits must be performed with numerical evaluation of the differential equations that define the theoretical models.

Likewise, the relationship between data and model can be complex, such as in the case when some parameters are shared across data sets while others are not. This is dealt with by utilizing a global analysis in which a simultaneous fit across all data is performed [8]. These scenarios typically arise from experiments repeated with most variables kept fixed, except for a few that vary. For instance, one might asses substance toxicity in bacteria by carrying out multiple experiments under varying concentration or type of toxic substances, but in otherwise fixed conditions [9]. To fit models to this data correctly, simultaneous analysis must be done, where parameters inherent to bacterial growth are kept fixed but substance-specific parameters are allowed to vary. Likewise, in models of amyloid aggregation [10], to elucidate aggregation mechanisms, simultaneous parameter fitting can be used to rule out certain mechanisms and provide evidence in support of others [11]. This can be achieved by varying a single variable between experiments and comparing potential theoretical models globally to the data [12]. The same is true for understanding bacterial growth dynamics [13], growth in mammals [14], the mitochondrial respiratory system [15], drug resistance [16], neural propagation [17], and many other biophysical systems.

In all of these scenarios, the application of standard fitting software tends to be limited and instead custom code must be developed. To allow efficient collaboration in such cases it can thus be necessary to develop graphical user interfaces or similar approaches to enable all collaborators to interact with the code. Moreover, these complex models are often not only difficult to implement, but also tend to be slow to fit; especially when there are many fit parameters to be determined. To speed up fitting procedures modern approaches such as using analytical gradient calculations (*“backpropagation”*) can be used, but these approaches have not seen broad adaption within biophysics yet.

## Implementation

In this short report, we present Simultant, a software application that allows complex functions to be fitted, potentially simultaneously across data sets, using a simple but general graphical user interface. The software allows custom complex functions or differentials equations to be specified as short Python snippets and automatically utilizes analytical gradient calculations to speed up fitting. A simple interface allows the specification of which functions and parameters belong to which data sets, and these can be easily shared across data. The software runs locally on any Windows, Mac or Linux machine. The code is open source and written in modern Javascript (electron–vue frontend) and Python (django–pytorch backend) and is thus easily extendable. Existing alternatives include AmyloFit [12] which is specialized for amyloid aggregation data and commercial fitting softwares OriginLab [18], GraphPad Prism [19] and KinTek Global Kinetic Explorer [20]. Compared with these, the interface of Simultant makes it simpler to define complex fitting setups, and in contrast, Simultant accelerates fitting using analytical gradient calculations, thus enabling large-scale fits to be performed. Finally, a major difference is that Simultant is open-source and thus easily extendable to custom needs.

## Results

**Fig. 1:**
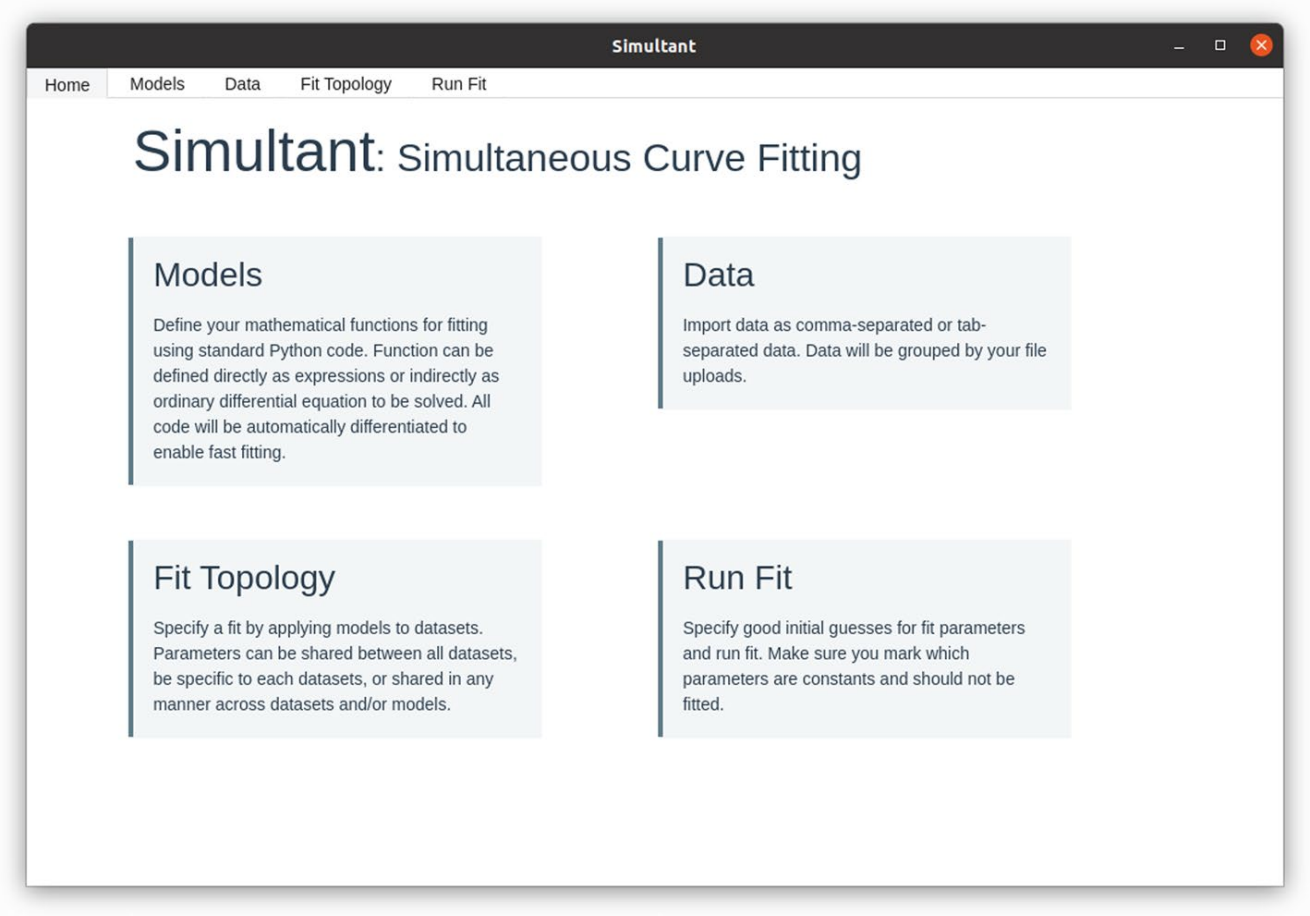
The welcome screen of Simulatant explains the four steps needed to specify and perform a fit

Using Simultant is a four step process as indicated in the main screen of the software (Fig. 1). You need to specify your (mathematical) models and upload data. Your models and data are saved in a database. You can then specify the specific fit topology: which models and parameters correspond to what data. Finally, you specify initial guesses for parameters and run the fit.

**Fig. 2:**
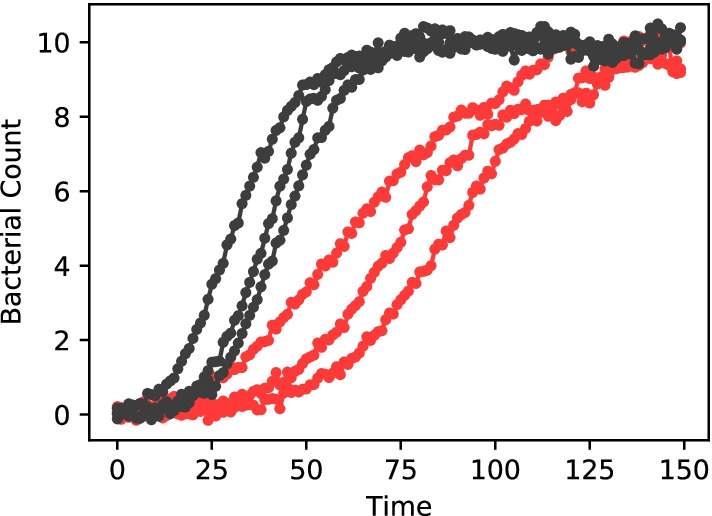
Synthetic bacterial growth curves. Data was generated using a Langevin equation of the form ${\mathrm{d}}N = r N \left[ 1 - ({N/K})^\nu \right] \, {\mathrm{d}}t + \sigma N {\mathrm{d}}W$ using carrying capacity *K* = 10, growth curvature $\nu = 0.5$, noise $\sigma = 0.01$, and growth rate *r* = 0.2 for the black curves and *r* = 0.1 for red curves. Initial conditions were varied for each run, $N(0) \in (0.01,\, 0.02,\, 0.1)$. Finally Gaussian ‘measurement’ noise was added with a standard deviation of 0.1

**Fig. 3:**
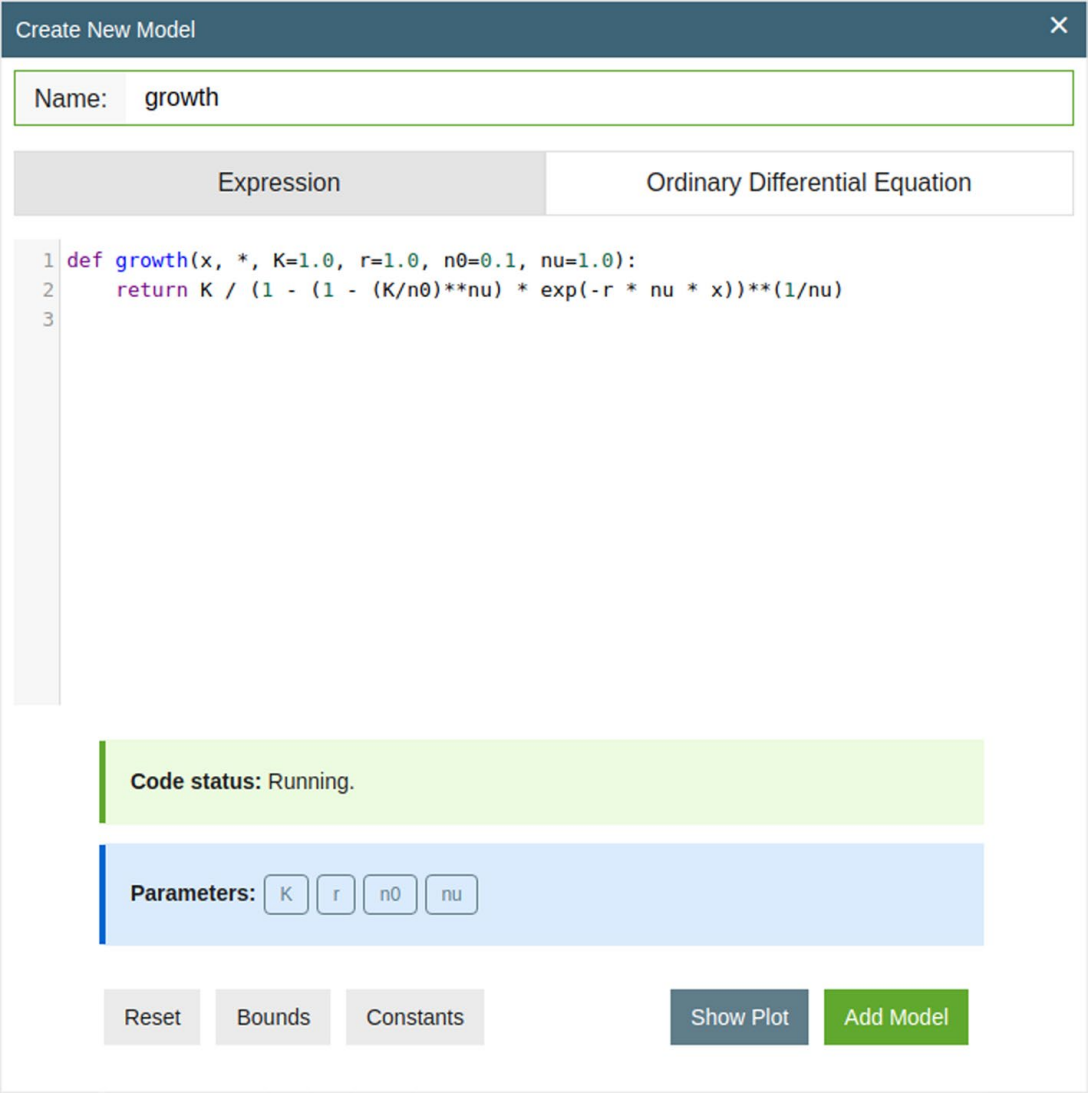
Adding model using an analytical expression. The syntax is standard Python. Note that arguments of the function are automatically recognized as fitting parameters. Constants and known bounds on the arguments can be specified using type annotations as described in the main text. Default values for the initial guesses are simply given as default arguments of the function variables

We will begin by exemplifying this process on a very simple, synthetic data set of bacterial growth. The data, shown and described in Fig. 2, was generated using a noisy generalized logistic growth model [21]. The data should thus approximately be described by

1 $$N(t) = K \left[ 1 - \left( 1 - \left( \frac{K}{N_0}\right) ^\nu \right) e^{-r \nu t} \right] ^{-{1/\nu }},$$

where *r* is the growth rate, *K* the carrying capacity, $\nu$ the growth curvature, and $N_0 = N(0)$ the initial bacterial concentration. In this case we have an analytical expression for the fitting function, and thus we can add it using a simple python function as shown in Fig. 3. The software automatically identifies function arguments as potential fitting parameters. Data is imported using .csv or .tsv files. Simply drag and drop files, or use the menu to select the data.

**Fig. 4:**
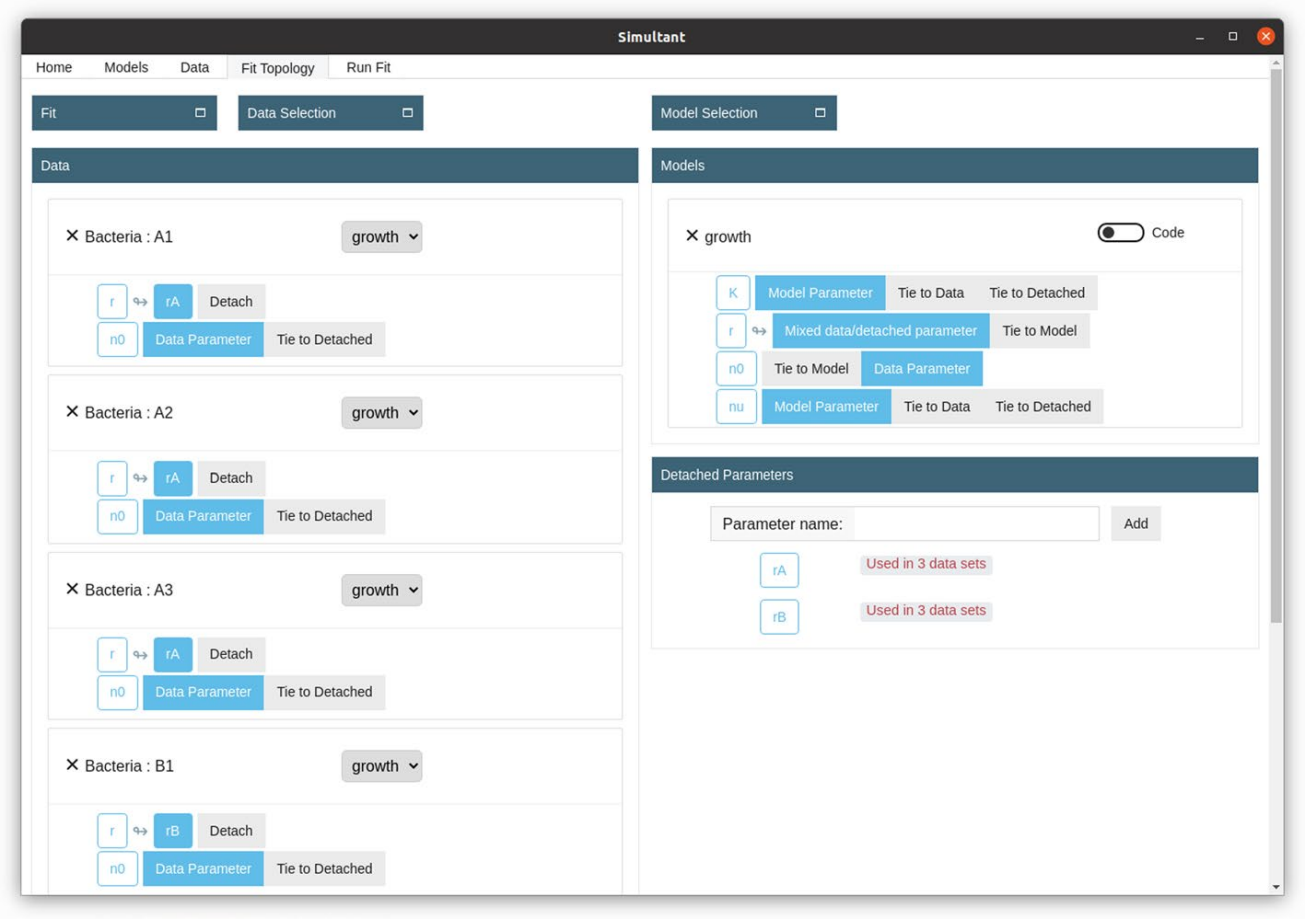
Fit topology. In our fit setup the parameters K and nu are *Model parameters* meaning their values is tied to the model and thus shared across all data sets that use the model. n0 is a *Data parameter* meaning that each data set has its own value of this parameter. Finally, the parameter r is tied to *Detached parameters* rA and rB such that half of the data sets uses rA and the other half uses rB. Parameters whose value are tied to a Detached parameter are indicated with an arrow. In total we have 10 free fitting parameters, six of which stem from n0 and two from nu

We now need to specify the fit topology. In the present case we have a single model (Eq. 1) that applies to all the data curves. In the section “Fit Topology” we select the data and add the model: when there is only one model chosen, it is automatically applied to all data sets. We then need to specify how the parameters are associated with the data sets. The typical approach to fitting data sets is to do one fit per data set, each with a free choice of parameters. In Simultant this corresponds to having each parameter set to the “Data parameter” type. However, in our present example only $N_0$ is independent for all data sets. The parameters *K* and $\nu$ are known to be the same across all data set and should thus be fitted simultaneously: this is achieved by choosing “Model parameter” for these parameters. Finally, the growth rate *r* is known to be shared across the two triplets of data sets shown in Fig. 2. We do this by defining “Detached parameters” and share them accordingly. This final setup in Simultant is shown in Fig. 4.

Finally we will run the fit. In the present example it is as simple as pressing “Run Fit”, but further adjustments could be needed: are some of the parameters constants that need not be fitted? Should some initial guesses of the parameters be changed? The software uses the limited memory Broyden–Fletcher–Goldfarb–Shanno (L-BFGS) algorithm with gradients calculated analytically. For fitting discontinuous models, the method can be changed to the Nelder–Mead algorithm, but this will in general be slower as it requires a lot more iterations to converge.

**Fig. 5:**
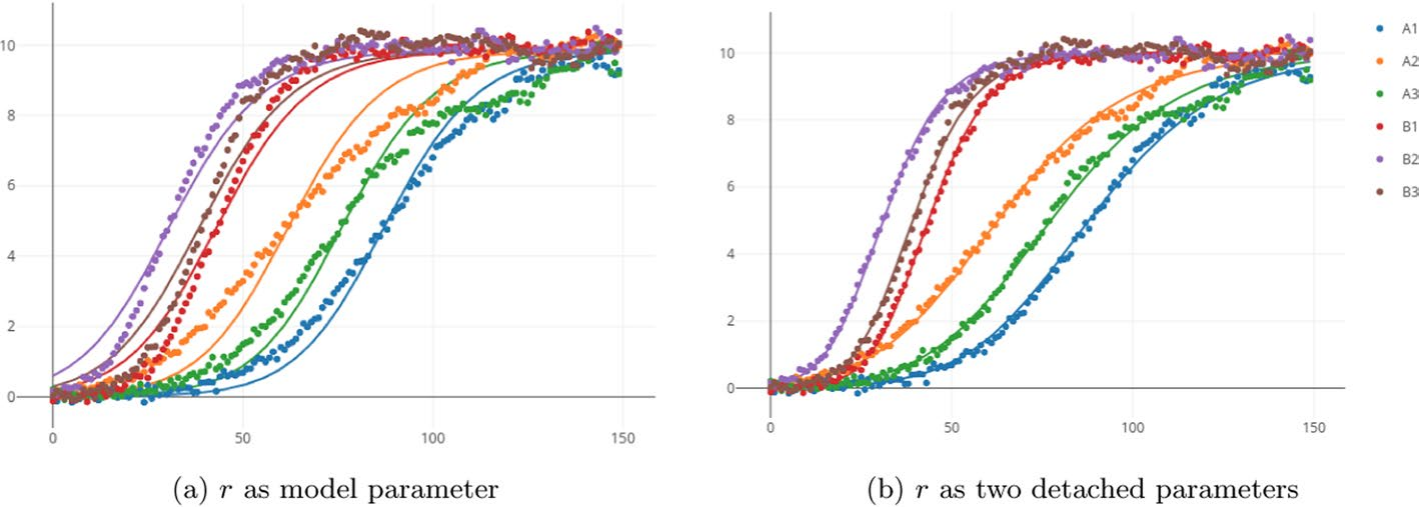
Simultaneous fits. **a** Using *r* as a model parameter does not result in a good overall fit. Even though the fit has $R^2 = 0.979$, it is clear that there are systematic errors. **b** Using two detached parameters results in excellent fits. This fit has $R^2 = 0.997$ which is comparable to the $R^2 = 0.998$ that can be obtained using completely independent fits (i.e. setting all parameters as data parameters). These plots were generated directly by Simultant

Figure 5 shows the final fit, both in the case where *r* is chosen to be a Model parameter (a) and the present case of *r* being tied two separate Detached Parameters. It is clear that the data cannot be described by a single growth rate *r*. Naturally, the data could easily be described if each curved was allowed a distinct *r*. Here we know that *r* should only take two values, one for each sub-data sets. Thus we use detached parameters and we see in Fig. 5b that our model is viable. Restricting the total number of parameters is key in distinguishing right from wrong in modeling [12].

As mentioned Simulatant can also define models indirectly via differential equations. This is done by specifying (Fig. 3) the input method as ‘Ordinary Differential Equation’ and then simply writing the ODE. For the present example this would be
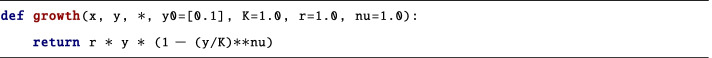


The rest of the process is exactly the same. However, it should be noted that ODE fitting is slower than expression fitting, and so it is important to choose good initial parameter guesses to speed up the process. The fact that Simulatant is able to do large-number-of-parameters ODE fitting at all is because it calculates gradients analytically. Using Nelder–Mead, or similar gradient free approaches, is significantly more time consuming for the present 10 parameter fit.

Simulatant allows the use of higher-order ODEs as well. These are simply specified with a function that returns more than one value. The GUI allows the specification of which dimension corresponds to the output of the fitting function. In more advanced cases a transform function can be defined, which defines the output as a custom function. Finally, event detection of the ODE is also possible in Simulatant, which can be used to e.g. normalize the ODE solutions by their steady state values.

Fitting is usually done with unconstrained parameters. However, often the mathematical model used implies certain restraints on the parameters. These constraint can be given to Simulatant as Python type hints. For example, the following function,  , has three parameters. The parameter ‘a’ is unbounded, parameter ‘b’ is positive only, and parameter ‘c’ is limited to the range [0, 1]. To avoid discontinuities at the boundaries and thus retain the ability to calculate gradients analytically, these bounds are implemented as parameter transforms. For example, for the parameter ‘b’, which is constrained to be positive, the fit is instead performed over a hidden variable ${\tilde{b}}$ which is unconstrained and defines $b = e^{{\tilde{b}}}$. A similar approach is used for interval constraints but using sigmoidal transform functions. Simulatant defaults parameters to being positive only. Not all parameters of a model are necessarily fitting parameters. To change the default type of a parameter to be constant, one may simply use C (for constant) instead of R (for range) in the type hint.

## Conclusions

In conclusion, Simulatant provides a simple user interface to design complex fitting setups. We have shown an elementary example use of Simulatant, where detached parameters were used to share some parameters between data sets. Detached parameters are more general than this as they can also be used to share parameters across models. Thus all possible combinations of data and models can be defined using this simple interface. Simulatant furthermore utilizes automatic gradient calculations which permits fast fitting even with many parameters. The software is furthermore easily extendable as the backend and frontend are completely separated and written in modern Python and Javascript. While the software is written using web technologies, the UI framework Electron allows this to run as a native application on Windows, Mac and Linux machines, but it can easily be hosted as a web server as well.

### Availability and requirements

- Project name: Simultant
- Project home page: https://github.com/juliusbierk/simultant
- Operating system(s): Platform independent
- Programming language: Python and Javascript
- License: MIT
- Any restrictions to use by non-academics: None

## Data Availability

The latest version of the software and its source code can be found at https://github.com/juliusbierk/simultant. A version has also been made available at Zenodo with 10.5281/zenodo.5541376.

## Appendix: Comparison to existing software

The following table makes a comparison between Simultant and other software typically used to perform fits of experimental data. As the underlying fitting procedures are similar, the fits that can be obtained with the software listed are all similar: what distinguishes them is the ease at which one can define a complex fitting problem, whether ODE fitting is possible, and whether they are commercial or not. We further note that most of the software listed have a much broader range of functionality than just fitting, but here we only compare on the features that Simultant provide: simultaneous expression/ODE fitting with automatic analytical gradient calculations.

Note that KinTek Global Kinetic Explorer [20] is specialized for analyzing the kinetics of chemical reactions, and AmyloFit [12] is specialized for analyzing amyloid aggregation data. The remaining software listed are generic in their applications.
